# Transcriptome Profiling of Atlantic Salmon Adherent Head Kidney Leukocytes Reveals That Macrophages Are Selectively Enriched During Culture

**DOI:** 10.3389/fimmu.2021.709910

**Published:** 2021-08-16

**Authors:** Nicole C. Smith, Navaneethaiyer Umasuthan, Surendra Kumar, Nardos T. Woldemariam, Rune Andreassen, Sherri L. Christian, Matthew L. Rise

**Affiliations:** ^1^Department of Ocean Sciences, Memorial University of Newfoundland, St. John’s, NL, Canada; ^2^Department of Life Sciences and Health, OsloMet-Oslo Metropolitan University, Oslo, Norway; ^3^Department of Biochemistry, Memorial University of Newfoundland, St. John’s, NL, Canada

**Keywords:** Atlantic salmon, transcriptome, macrophage, microarray, cell differentiation, head kidney leukocyte culture

## Abstract

The Atlantic salmon (*Salmo salar*) is an economically important fish, both in aquaculture and in the wild. In vertebrates, macrophages are some of the first cell types to respond to pathogen infection and disease. While macrophage biology has been characterized in mammals, less is known in fish. Our previous work identified changes in the morphology, phagocytic ability, and miRNA profile of Atlantic salmon adherent head kidney leukocytes (HKLs) from predominantly “monocyte-like” at Day 1 of *in vitro* culture to predominantly “macrophage-like” at Day 5 of culture. Therefore, to further characterize these two cell populations, we examined the mRNA transcriptome profile in Day 1 and Day 5 HKLs using a 44K oligonucleotide microarray. Large changes in the transcriptome were revealed, including changes in the expression of macrophage and immune-related transcripts (e.g. *csf1r, arg1, tnfa, mx2)*, lipid-related transcripts (e.g. *fasn, dhcr7, fabp6*), and transcription factors involved in macrophage differentiation and function (e.g. *klf2, klf9, irf7, irf8, stat1*). The *in silico* target prediction analysis of differentially expressed genes (DEGs) using miRNAs known to change expression in Day 5 HKLs, followed by gene pathway enrichment analysis, supported that these miRNAs may be involved in macrophage maturation by targeting specific DEGs. Elucidating how immune cells, such as macrophages, develop and function is a key step in understanding the Atlantic salmon immune system. Overall, the results indicate that, without the addition of exogenous factors, the adherent HKL cell population differentiates *in vitro* to become macrophage-like.

## Introduction

Macrophages are white blood cells, found in all vertebrate species, that play a role in both the innate and adaptive immune systems (1). In innate immunity, macrophages provide some of the first lines of defense against infections and diseases, where they act as phagocytic cells to destroy foreign pathogens (2). In the adaptive immune system, macrophages function as a bridge between the innate and adaptive immune responses, acting as antigen-presenting cells to activate T lymphocytes (2, 3). Much of our knowledge of macrophage biology, such as macrophage differentiation and polarization, comes from mammalian models, while macrophages remain to be fully characterized across all fish species. However, using the mammalian model system as a platform and through various fish models, including zebrafish (*Danio rerio*), ginbuna crucian carp (*Carassius langsdorfii*) and goldfish (*Carassius auratus*), our knowledge of fish macrophage differentiation and activation is starting to expand [reviewed in (4)].

The ways in which macrophages respond to infections and diseases have been well-characterized in mammals: by producing cytokines and other inflammation-related proteins, by engulfing foreign pathogens through phagocytosis, and by destroying foreign pathogens by producing reactive oxygen species (ROS) and nitric oxide (NO), among other responses (2, 5). Macrophages demonstrate a high degree of plasticity, with the ability to generate different subtypes (also well described in mammals): M1 macrophages (or classically activated) and M2 macrophages (or alternatively activated) (6). M2 macrophages can be further separated into distinct sub-populations, based on their activation and function (M2a, M2b, M2c) (6). M1 macrophages are considered pro-inflammatory; they are activated by cytokines including IFN-γ and TNF-α and produce pro-inflammatory cytokines and ROS to protect against pathogens (7). Similar to mammals, IFN-γ and TNF-α have been described in several fish species, where they induce pro-inflammatory effects including increased phagocytosis, increased ROS and NO production, and enhanced expression of inflammatory cytokines (8–16). On the other hand, M2 macrophages are considered anti-inflammatory and are linked to immunosuppression and wound repair. M2 macrophages are activated by cytokines such as IL-4 and IL-13 (M2a), immune complexes or apoptotic cells (M2b) and IL-10, TGF-β or glucocorticoids (M2c) and are characterized by increased arginase activity, decreased microbicidal activity, and increased production of collagen and polyamines necessary for cell growth and wound-healing (3, 7, 17, 18). Teleost fish *il-4/13A* and *il-4/13B* genes have been identified and have similar functions as their mammalian counterparts; stimulation of macrophages from various teleost species with recombinant (r-) IL-4/13A and r-IL-4/13B increased the expression of immunosuppressive genes such as *tgf*-*β*, *il-10* and *socs3*, increased arginase activity, and decreased the expression of pro-inflammatory genes and NO production (3, 10, 18–21).

Hematopoiesis, the process of blood cell formation, begins when a self-renewing hematopoietic stem cell (HSC) commits to a multipotent progenitor (MPP), which then gives rise to a common myeloid progenitor (CMP) cell. The CMP will then differentiate into either a megakaryocyte/erythroid progenitor (MEP) or a granulocyte/macrophage progenitor (GMP), which gives rise to erythrocytes/platelets or granulocytes/monocytes, respectively (4, 22). This process is tightly controlled by a multitude of cytokines, growth factors, and transcription factors and has been extensively studied in mammals. In particular, monocyte-to-macrophage differentiation, as well as macrophage polarization, are regulated by multiple factors including the growth factor colony-stimulating factor 1 (CSF1) and its receptor, CSF1R, the transcription factor PU.1, and members of the CCAAT/enhancer-binding proteins (C/EBP), interferon regulatory factor (IRF) and signal transducer and activator of transcription (STAT) families, among many others [reviewed in (23)]. One of the first studies to investigate fish macrophage differentiation examined goldfish primary kidney macrophages and identified three sub-populations that were characterized as progenitor cells, monocytes, and macrophages, with each population expressing differentiation markers including *c-kit* (early progenitors), *granulin* (monocytes) and *legumain* (mature macrophages) (24). It is now well-known that CSF1 and CSF1R are required for both mammalian and teleost myeloid cell differentiation (3). While our knowledge of fish macrophage biology is advancing, macrophage differentiation and polarization across all teleost species, including the Atlantic salmon (*Salmo salar*), remain to be described. The Atlantic salmon is an economically important farmed fish species in several countries including Canada, Norway and Chile. Given the essential role of macrophages in defense against pathogens, investigation into the genes and molecular pathways involved in Atlantic salmon macrophage differentiation and function is central to fully understanding the fish immune response and will aid in developing methods of disease prevention, therefore improving the health of farmed fish.

In mammals, HSCs originate from the bone marrow, while in fish, the primary hematopoietic organ is the anterior (or head) kidney. A heterogeneous population of adherent leukocytes, containing monocytes and macrophages, amongst other cells, can be isolated from the head kidney using Percoll density gradient centrifugation (21, 25, 26). Adherent head kidney leukocytes (HKLs) are frequently used as a macrophage-like model in fish immunological studies [(27–31), and many others]; however, many of these studies use HKLs from different culture times, which may produce data that are from different cell populations. Our previous work observed a change in the morphology, phagocytic ability, and miRNA profile of Atlantic salmon HKLs *in vitro*, suggesting that the cells differentiate from predominantly “monocyte-like” at Day 1 of culture to predominantly “macrophage-like” at Day 5 of culture (32). Microarrays are powerful tools that have been used to identify changes in gene expression profiles during fish immune responses [reviewed in (33, 34)]. Therefore, to further characterize the HKLs *in vitro*, we used 44K salmonid oligonucleotide microarrays (35) to examine the global transcript expression profiles of Atlantic salmon adherent Day 1 HKLs versus Day 5 HKLs.

## Materials and Methods

### Animals

The Atlantic salmon (1.2 kg ± 0.3 kg SD) used in this experiment were held in the Dr. Joe Brown Aquatic Research Building (JBARB) of the Ocean Sciences Centre in a 3,800 L tank and kept at 12°C with 95-110% oxygen saturation, using a flow-through seawater system. All procedures in this experiment were approved by Memorial University of Newfoundland’s Institutional Animal Care Committee (protocols: 18-01-MR and 14-02-MR) based on the guidelines of the Canadian Council of Animal Care. Five animals were used for the microarray experiment (one animal was removed following array hybridizations due to a technical error in labelling, therefore 4 animals were used for subsequent analysis), and 5 different animals were used for RT-qPCR analysis.

### Macrophage Isolation and Culture

HKLs were isolated as previously described (32, 36). Atlantic salmon were euthanized with an overdose of MS222 (0.4 g/L, Syndel Laboratories, Vancouver, BC, Canada). The head kidney was removed and placed in isolation media: 500 mL of Leibovitz-15 medium (L-15, Gibco, Carlsbad, CA, USA) supplemented with 2.5% fetal bovine serum (FBS, Gibco), 1% penicillin/streptomycin (Gibco), and 27.5 mg of heparin (Sigma-Aldrich, St. Louis, MO, USA). The head kidney was pushed through a 100 µM nylon cell strainer (Thermo-Fisher Scientific, Waltham, MA, USA), then placed on a 34/51% Percoll gradient (GE Healthcare, Uppsala, Sweden) prepared with 5% Hank’s buffered salt solution (HBSS; Sigma-Aldrich) to ensure an isotonic solution, and centrifuged at 500 x g for 30 min at 4°C. Following centrifugation, the interface between the 34% and 51% gradient, which contains leukocytes, was collected and washed twice in isolation media at 500 x g for 5 min at 4°C. The cells were then re-suspended in culture media (L-15 supplemented with 5% FBS and 1% penicillin/streptomycin), and viable cells were counted on a hemocytometer using the Trypan Blue (Sigma-Aldrich) exclusion method. The cells were then seeded in 6-well culture plates (Corning Inc., Corning, NY, USA) at 3 x 10^7^ cells in 2 mL of culture media and incubated at 15°C for 24 h to allow cell adherence. Cells were then washed twice in culture media to remove non-adherent cells, and the media was replaced with fresh culture media. Media was changed every 48 h thereafter for up to 5 days.

### Sampling of Head Kidney Cells for RNA Extraction

Twenty-four hours (Day 1) and 120 h (Day 5) after seeding, cells were washed twice in cell culture media then lysed in 500 μL of TRIzol (Invitrogen, Burlington, ON, Canada) and immediately placed at -80°C until RNA extraction.

### RNA Extraction

Total RNA was extracted from the TRIzol-lysed samples following the manufacturer’s protocol, and RNA pellets were dissolved in DNase/RNase-free water (Gibco). The RNA samples were treated with 6.8 Kunitz units of DNase I (Qiagen, Mississauga, ON, Canada) to degrade residual genomic DNA, followed by purification using the RNeasy MinElute Cleanup Kit (Qiagen) according to the manufacturer’s protocol. RNA concentration was measured using NanoDrop spectrophotometry, and RNA integrity was checked by 1% agarose gel electrophoresis. All column-purified RNA samples had A260/280 and A260/230 ratios above 1.8.

### Microarray Hybridization

Day 1 (24 h) and Day 5 (120 h) samples were subjected to microarray analysis using the consortium for Genomic Research on All Salmonids Project (cGRASP)-designed Agilent 44K salmonid oligonucleotide microarray (35). The microarray experiment was based on a common reference design, where the differences among Day 1 and Day 5 HKL samples were determined by comparing individual samples against a common reference pool consisting of equal quantities from all samples.

**Figure 1:**
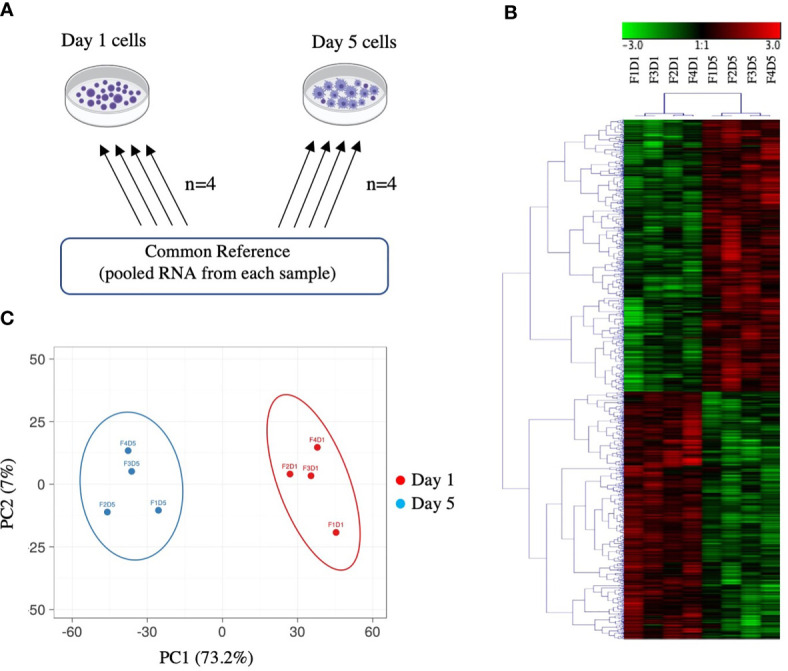
Overview of microarray experimental design and global gene expression profiles. **(A)** Common reference-based microarray experimental design. Each arrow represents one array and identifies the samples co-hybridized on that array; the base of the arrow identifies the Cy3-labeled sample and the head of the arrow identifies the Cy5-labeled sample. **(B)** Hierarchical clustering analysis of 2140 DEPs in Day 1 and Day 5 HKLs identified by paired SAM (FDR 0.05). Complete linkage was performed on median-centred genes using a Pearson correlation. Green represents downregulation and red represents upregulation. F represents fish; D represents Day (i.e. F1D1 is Fish 1 Day 1). **(C)** Principal component analysis (PCA) of Day 1 and Day 5 samples based on DEPs identified by paired SAM (FDR 0.05). Day 5 samples are represented by blue, Day 1 samples are represented by red. The X and Y axis show principal component 1 (PC1) and principal component 2 (PC2) that explain 73.2% and 7% of the total variance, respectively.

Five hundred nanograms of each sample of DNase-treated, column purified RNA were *in vitro* transcribed into antisense amplified RNA (aRNA) using the Amino Allyl MessageAmp™ II aRNA Amplification Kit (Ambion, Carlsbad, CA, USA) following the manufacturer’s instructions. The quality and quantity of the aRNAs were checked by agarose gel electrophoresis and NanoDrop spectrophotometry, respectively. Amplified RNA from all samples was pooled and used as a common reference. Twenty micrograms of aRNA were ethanol precipitated overnight and re-suspended in coupling buffer. The experimental samples were then labelled with Cy5 (GE Healthcare Life Sciences, Buckinghamshire, UK), while the common reference was labelled with Cy3 (GE Healthcare Life Sciences), following the manufacturer’s instructions. The efficiency of labelling and aRNA concentration were assessed using the “microarray” function of the NanoDrop spectrophotometer. The Cy5-labelled aRNA (825 ng) from each experimental sample was mixed with an equal quantity of Cy3-labelled aRNA from the common reference, and the resulting pool was fragmented using the Gene Expression Hybridization Kit, following the manufacturer’s instructions (Agilent, Mississauga, ON, Canada). Each labelled aRNA pool was co-hybridized to the microarray (8 arrays final in total, **Figure 1A**) for 17 h at 65°C with 10 rpm rotation using an Agilent hybridization oven. The array slides were washed immediately following hybridization as per the manufacturer’s instructions.

### Microarray Data Acquisition and Analysis

The microarray slides were scanned at 5 µm resolution and 90% laser power using a ScanArray Gx Plus scanner and ScanExpress v4.0 software (Perkin Elmer, Waltham, MA, USA), and the Cy3 and Cy5 channel photomultiplier tube (PMT) settings were adjusted to balance the fluorescence signal. The raw data were saved as TIFF images, and the signal intensity data were extracted using Imagene 9.0 (BioDiscovery, El Segundo, CA, USA). R and the Bioconductor package ‘marray’ were used for background correction, removal of low-quality spots on the microarray and to log_2_-transform and Loess-normalize the data (37). Probes with more than 25% missing values were omitted from the dataset, and the missing values were imputed using the least square methods (‘EM_array’) and the ‘LSimpute’ package (37–39). The final dataset that was used for statistical analyses consisted of 18,108 probes for all arrays. The data have been submitted to NCBI’s Gene Expression Omnibus and are accessible through GEO Series accession number GSE173493.

A two-class paired Significance Analysis of Microarrays (SAM) (40) with a false discovery rate (FDR) of 0.05 was used to determine the differentially expressed probes (DEPs) between Day 1 and Day 5 groups, using R and the SAM project GitHub repository (https://github.com/MikeJSeo/SAM) (41). The resulting significant transcript lists were annotated using the contiguous sequences that were used to design the 60mer oligonucleotide probes of the array (35). Annotation was carried out with BLASTx searches against the NCBI non-redundant (nr) amino acid sequence database using an E-value threshold of 10^-5^ (42).

### GO Term Enrichment and Network Analysis, Hierarchical Clustering Analysis and Principal Coordinate Analysis

Gene Ontology (GO) term enrichment analyses for all (both upregulated and downregulated) differentially expressed genes (DEGs; the distinction between DEPs and DEGs is explained in section 3.1), with a fold-change > |2| were performed using ClueGO plugin, available for the Cytoscape software (version 3.8.2). The ClueGO plug-in identifies and integrates significant GO terms from large gene lists and generates a functionally grouped GO term network (43). In this study, the GO database (30.03.2021) for the categories biological process (BP) and cellular component (CC) was used for analysis. The enrichment/depletion analysis was performed using a two-sided hypergeometric test after its adjustment by the Bonferroni step-down procedure. The kappa-statistics score threshold was set to 0.4 and GO pathways/terms with a p-value <0.05, corrected with the Bonferroni step-down procedure, were considered significant.

Genesis software (Rockville, MD, USA) was used for the hierarchical clustering and heatmap visualization of median centered data of DEPs (for analysis of the entire experiment) and of DEGs (for analysis of selected significant GO terms identified using ClueGO; see section 3.2) using Pearson correlation and complete linkage clustering. Hierarchical clustering analysis of all DEPs grouped Day 1 samples together and Day 5 samples together, with the exception of 1 fish (Fish 5), which we eliminated from further analysis. Principal components were calculated using the Singular Value Decomposition method and ClustVis: a web tool for visualizing clustering of multivariate data using Principal Component Analysis and heatmap (44).

### cDNA Synthesis

Five hundred nanograms of purified RNA were reverse transcribed to cDNA in 20 µL reactions consisting of random primers (250 ng; Invitrogen) and MMLV-reverse transcriptase (200 U; Invitrogen) with the manufacturer’s first-strand buffer (1x final concentration), DTT (10 mM final concentration), 10 mM dNTP mix (10 mM each of dATP, dGTP, dCTP and dTTP) and RNase OUT (40 Units; Invitrogen) at 37°C for 50 min.

### Reverse Transcription Quantitative PCR (RT-qPCR)

For RT-qPCR validation, HKLs from 5 additional Atlantic salmon (i.e. different from those used in the microarray experiment) were harvested, RNA isolated, and cDNA synthesized as described above. All primer sets used for RT-qPCR analysis were quality-tested according to MIQE guidelines (45). For each primer set, amplification efficiencies were determined by a 5-point standard curve using pooled cDNA from 5 fish, starting at 10 ng of input RNA, diluted in DNAse/RNAse-free water (Thermo Fisher Scientific) (46). Only primer pairs generating an amplicon with a single melting peak and no primer-dimer present in the no-template control (NTC) were used for RT-qPCR analysis. Primer sequences, amplification efficiencies, R^2^, and amplicon sizes for each assay can be found in **Supplementary Table 1**.

Five candidate normalizer genes were tested with cDNA from all experimental samples to determine the 2 most stable normalizer transcripts (i.e. with lowest M-value) using GeNorm software (47). The candidate normalizer genes tested were *60S ribosomal protein 32 (rpl32), elongation factor 1 alpha-1 (ef1a1)*, *RNA polymerase 2 (polr2)*, *polyadenylate-binding protein 1 (pabpc1) and elongation factor 1-alpha-2 (ef1a2)*. The 3 most stable genes were *ef1a2* (M-value 0.180), *ef1a1* (M-value 0.187) and *rpl32* (M-value 0.198). The normalizers chosen for this study were *ef1a2* and *rpl32*. The geometric mean of *ef1a2* and *rpl32* was calculated for each sample and was used as normalizer value in the relative quantity (RQ) calculations stated below.

For each reaction, 50 nM of both the forward and reverse primers and cDNA template representing 5 ng of input RNA were mixed with Power SYBR Green Master Mix (Thermo Fisher Scientific) for a total reaction volume of 13 µl. The real-time analysis program consisted of 1 cycle of 50°C for 2 min, 1 cycle of 95°C for 10 min, and 40 cycles of 95°C for 15 sec and 60°C for 1 min, with fluorescence detection at the end of each 60°C step. All reactions were run in triplicate in a ViiA 7 Real-Time PCR System (384-well format) (Applied Biosystems/Life Technologies). The RQ values of a given mRNA of interest were calculated using Excel, and relative to a calibrator [i.e. the Day 1 sample with the lowest expression (i.e. assigned a RQ value = 1.0)] taking into account the amplification efficiencies (46). A paired Student’s T-test was used to determine statistical differences. Differences were considered statistically significant at P<0.05. All statistical analyses were performed using GraphPad Prism v 8.0 (GraphPad Software, La Jolla, CA, USA, www.graphpad.com).

### *In Silico* Prediction of Putative miRNA Target Genes and Target Gene Pathway Analysis

The miRNA target prediction tool RNAhybrid (v.2.2) (48) was used to determine if any of the DEGs identified in this study could be potential targets of the miRNAs identified as significantly differentially expressed (DE) in Day 1 monocyte-like cells compared with Day 5 macrophage-like cells in Smith et al. (32). The mature miRNAs analyzed were selected from those DE in Smith et al. (32), but in cases where both mature miRNAs from the same precursor were DE then only the most abundant (which is most likely to be the guide miRNA) was used. The 36 miRNAs used, along with their mature sequences (49) are given in **Supplementary Table 2**. The parameters applied in the RNA hybrid analysis were: No G:U in seed, helix constraint 2–8, loop constraints 5–5 and a minimum free energy threshold of -20 kcal/mol. These parameters allowed RNAhybrid to detect only candidate genes with perfect seed complementarity and high base-pairing stability.

The input sequences for target genes were those DEGs from this study with 3’ untranslated region (UTR) information, found using the ExUTR pipeline (50); i.e. a total of 1234 out of the 1477 DEGs. The predicted target genes from the *in silico* target gene prediction analysis were used as input in a gene pathway enrichment analysis (51) against the bioplanet database of all known biological pathways (52). Gene pathways are poorly described in Atlantic salmon, therefore the gene symbols for putative human orthologs were used against the human database. The significance level for enrichment was set as p-adjusted (Q-value) less than 0.05.

## Results

### Global Transcriptomic Changes in Atlantic Salmon HKLs in Response to Culture Period

Our previous work identified a change in the morphology, phagocytic ability, miRNA profile, and mRNA expression of two macrophage markers (*mhc ii* and *marco*), in Day 1 and Day 5 adherent HKLs (32). To explore changes in the mRNA transcriptome between these two cell populations, the DEPs between Day 1 (i.e. predominantly monocyte-like) and Day 5 (i.e. predominantly macrophage-like) HKLs were identified using a 44K salmonid microarray platform (35). The design for this microarray study is illustrated in **Figure 1A**. Using paired Significance Analysis of Microarrays (SAM) and a false discovery rate (FDR) of 0.05, 2140 DEPs were identified; 1123 DEPs were identified as upregulated in Day 5 HKLs compared to Day 1 HKLs while 1017 DEPs were downregulated in Day 5 HKLs compared to Day 1 HKLs. Using BLASTn/BLASTx searches against NCBI nr/nt databases, putative identities were determined for 2034 of the 2140 DEPs (1076 upregulated DEPs, 958 downregulated DEPs). The 44K platform contains some redundancies (i.e. multiple probes for one gene). Therefore, taking the redundancy into account, 1477 differentially expressed genes (DEGs) with known putative identities were identified (797 upregulated DEGs in Day 5 and 680 downregulated DEGs in Day 5). Selected DEPs for discussion can be found in **Table 1**, and complete information on the DEPs and paired SAM results can be found in **Supplementary Tables 3** and **4**.

**Table 1:** Selected^a^ probes differentially expressed between Day 1 and Day 5 HKLs.

Upregulated in Day 5 HKLs				
	Probe ID^b^	Gene symbol	Gene description^c^	Log_2_ fold-change^d^
**Immune-related**				
	C228R013	*tlr3*	Toll-like receptor 3_1_*	4.04
	C157R134	*csf1r*	Macrophage colony-stimulating factor 1 receptor_4_	3.28
	C095R005	*il12b*	Interleukin-12 subunit beta_2_	3.12
	C040R101	*ifit5*	Interferon-induced protein with tetratricopeptide repeats 5_4_	2.86
	C163R118	*mrc1*	Macrophage mannose receptor 1_5_	2.71
	C236R043	*mx2*	Interferon-induced GTP-binding protein Mx2_2_	2.57
	C237R068	*tnfa*	Tumor necrosis factor (TNF-alpha)_2_	2.45
	C041R022	*mx3*	Interferon-induced GTP-binding protein Mx3_1_	2.28
	C022R023	*socs1*	Suppressor of cytokine signaling 1_1_	2.18
	C139R032	*rsad2*	Radical S-adenosyl methionine domain-containing protein 2 (alias viperin)_1_	2.13
	C029R132	*ifng1*	Interferon gamma 1_2_	2.06
	C198R010	*hamp*	Hepcidin-1_2_	1.94
	C063R127	*ddx58*	Probable ATP-dependent RNA helicase DDX58_2_	1.59
	C174R152	*cd83*	CD83 antigen_5_	1.30
**Lipid-related**				
	C066R040	*fadsd5*	Delta-5 fatty acyl desaturase_1_	5.78
	C227R073	*lpl*	Lipoprotein lipase_2_	4.45
	C193R045	*elovl6*	Elongation of very long chain fatty acids protein 6_1_	4.19
	C180R145	*lipe*	Lipase, hormone-sensitive_3_*	3.99
	C038R110	*fadsd6*	Delta-6 fatty acyl desaturase (alias fatty acid desaturase 2 (*fads2)*)_1_*	3.79
	C119R039	*dhcr7*	7-dehydrocholesterol reductase_2_	3.26
	C004R046	*fasn*	Fatty acid synthase_3_	3.11
**Transcription**				
**factors**	C261R073	*stat1*	Signal transducer and activator of transcription 1-alpha/beta_3_*	2.73
	C143R078	*irf7*	Interferon regulatory factor 7_3_ ^e^	2.70
	C169R001	*irf3*	Interferon regulatory factor 3_1_ ^e^	2.30
	C169R089	*irf8*	Interferon regulatory factor 8_3_ ^e^	1.26
**Downregulated in Day 5 HKLs**				
**Immune-related**				
	C056R147	*tnfrsf6b*	Tumor necrosis factor receptor superfamily member 6B_2_	-3.95
	C233R142	*cfd*	Complement factor D_2_	-3.20
	C157R080	*cd79a*	B-cell antigen receptor complex-associated protein alpha chain_1_	-3.06
	C249R147	*cd28*	T-cell-specific surface glycoprotein CD28_1_	-2.84
	C158R168	*btla*	B- and T-lymphocyte attenuator_1_	-2.76
	C121R047	*tnfrsf11b*	Tumor necrosis factor receptor superfamily member 11B_2_	-2.56
	C252R066	*cxcr4*	C-X-C chemokine receptor type 4-A_2_	-2.46
	C017R011	*csf3r*	Granulocyte colony-stimulating factor receptor_1_	-2.43
	C162R124	*cxcr1*	C-X-C chemokine receptor type 1-like_2_*	-2.42
	C203R099	*ighm*	Ig heavy chain Mem5_15_	-2.37
	C206R019	*tlr9*	Toll-like receptor 9_1_	-2.10
	C249R147	*cd28*	T-cell-specific surface glycoprotein CD28_5_	-2.00
	C241R142	*arg1*	Arginase-1_2_*	-1.99
	C230R100	*il1b*	Interleukin 1 beta_1_	-1.56
	C251R068	*tgfb1*	Transforming growth factor beta-1 proprotein_1_	-1.21
**Lipid-related**				
	C211R005	*fabp6*	Fatty acid binding protein 6 (alias gastrotropin)_3_	-4.75
	C043R091	*alox5ap*	Arachidonate 5-lipoxygenase-activating protein_1_	-1.40
**Transcription**				
**Factors**	C259R111	*klf2*	Krueppel-like factor 2_2_	-4.02
	C055R098	*jun*	Transcription factor AP-1 (alias jun proto-oncogene)_1_	-3.60
	C142R114	*klf9*	Krueppel-like factor 9_1_	-2.18
	C088R028	*runx3*	Runt-related transcription factor 3-like_1_*	-1.61

a Probes were selected based on their known immune-related function and/or immune response in both fish and mammalian literature. See **Supplementary Table 3** for complete list of differentially expressed probes.

b 44K microarray identifier. When multiple probes share the same annotation, the probe ID with the largest log_2_ fold-change was indicated.

c Taken from the most significant (lowest E-value) BLASTx hit in the Blast2GO annotation. If no reliable BLASTx hits were found, the best BLASTn hit was chosen instead and is represented by an asterisk (*). If BLASTn and BLASTx analyses for a given probe showed different results, then the best BLASTn hit was reported. The subscript after the BLASTx hit’s name represents the number of differentially expressed probes sharing the same annotation.

d Log_2_ fold-change (Day 5/Day 1) for differentially expressed probes (FDR < 0.05) as determined by SAM analysis. An average log_2_ fold-change was taken when multiple probes with the same annotation were differentially expressed.

e Transcription factors that are also immune-relevant.

Hierarchical clustering analysis of median-centered DEPs grouped Day 1 samples and Day 5 samples separately (**Figure 1B**). Similarly, principal component analysis (PCA) also grouped Day 1 samples separately from Day 5 samples together (**Figure 1C**). PC1 and PC2 accounted for 73.2% and 7.0% of the variation, respectively. Day 1 samples showed a positive loading on PC1, whereas Day 5 samples showed a negative loading on PC1. There was a near split between positive/negative loading on PC2 with both Day 1 and Day 5 samples. These data indicate that Day 1 HKLs and Day 5 HKLs represent two separate groups of cells with distinct molecular phenotypes.

### GO Term Network Analysis Identified Immune-Related and Lipid-Related Terms

**Figure 2:**
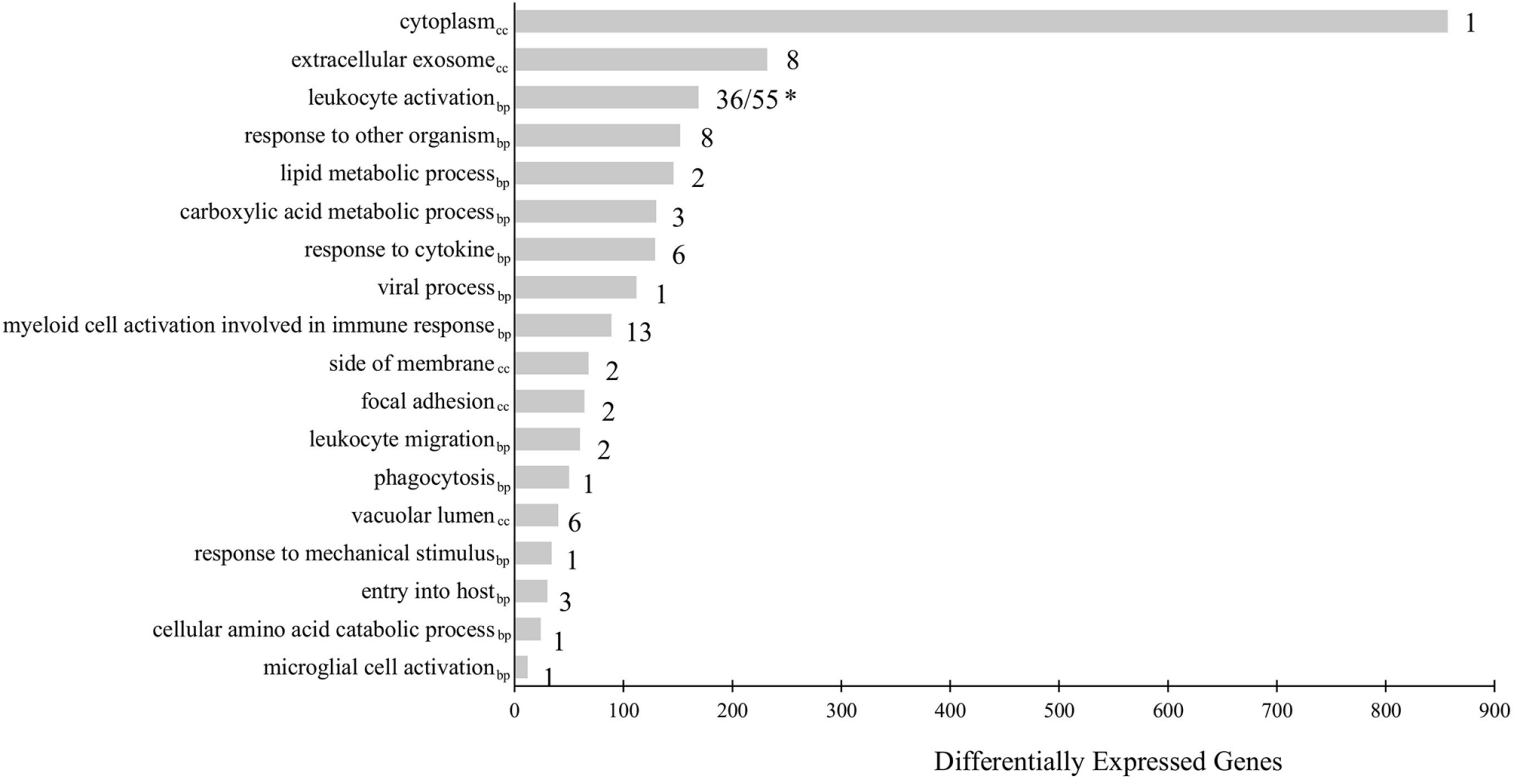
Gene Ontology (GO) term enrichment analysis of all differentially expressed genes (DEGs) between Day 1 and Day 5 HKLs with a fold-change > |2|. The leading term of each identified group is shown. The bars represent the number of DEGs associated with the term while the number after each bar represents the number of GO terms associated with that group. * “Leukocyte activation” is the leading term for two groups: Group 17 (consisting of 36 GO terms) and Group 18 (consisting of 55 GO terms). GO terms from the Biological Process database are identified by the subscript “bp”, while GO terms from Cellular Component are identified by “cc”.

**Figure 3:**
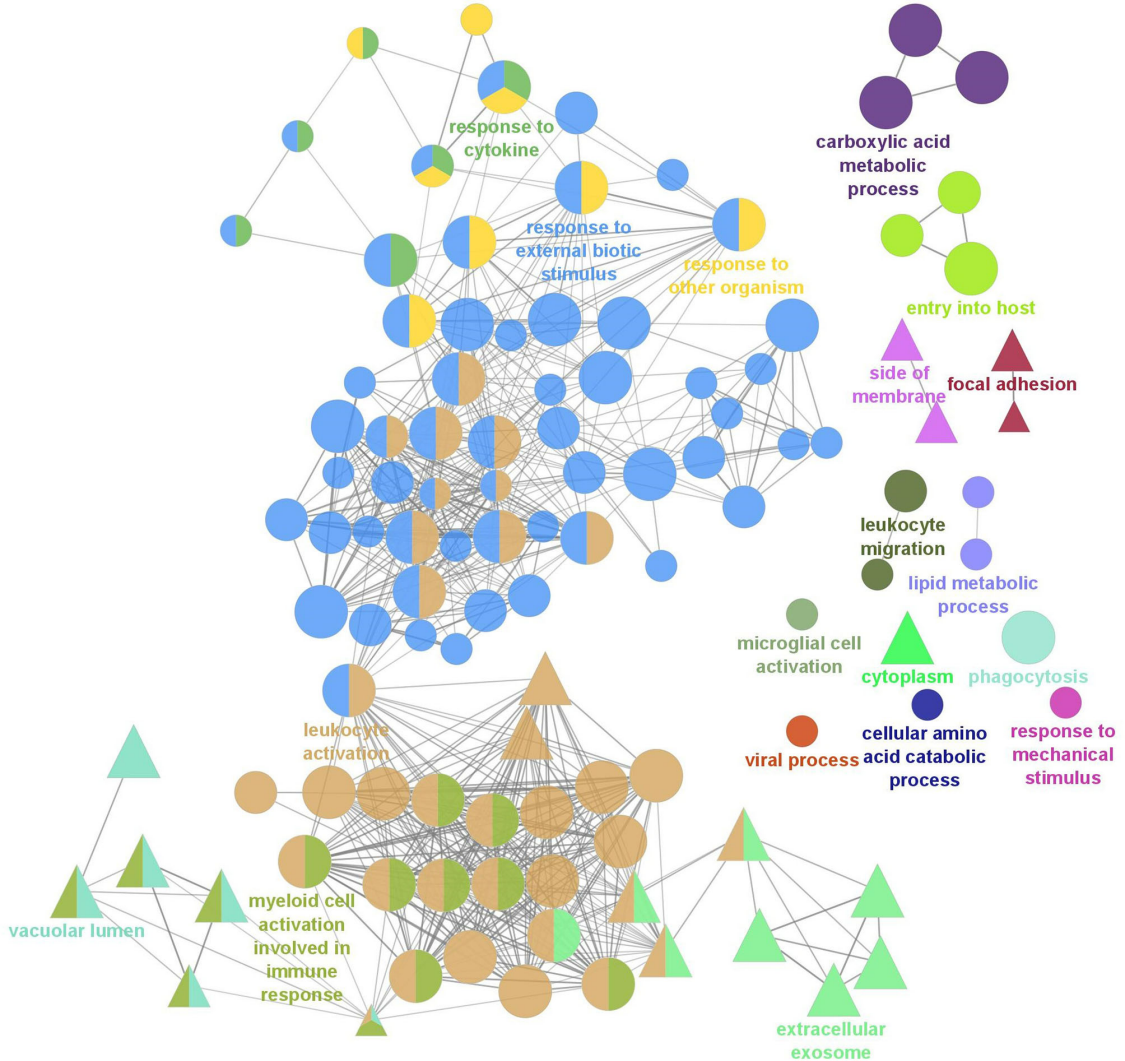
Gene Ontology (GO) term enrichment and network analysis of all DEGs between Day 1 and Day 5 HKLs. Two GO databases were used, Biological Process (BP; represented by circles) and Cellular Component (CC; represented by triangles) and each node represents a significantly enriched GO term (p<0.05, corrected with the Bonferroni step-down procedure). Related GO terms are labelled with the same colour and, when a term is shared by two or more GO cluster groups, the node is illustrated by multiple colours. The most significant terms unique to BP and CC are labelled. The size of the node represents the enrichment significance of the terms, and the thickness of edges indicates the kappa score.

To further understand the biological relevance of the identified DEGs, gene ontology (GO) term enrichment analyses, followed by network analysis, were performed on all DEGs with a fold-change greater than |2| (FDR = 0.05). GO terms with p-values less than 0.05 were considered statistically significant. The analysis resulted in 111 significant GO terms divided into 19 groups. The top GO term group (i.e. lowest individual term p-value) was “leukocyte activation” (GO:0045321; p-value 1.34e-15) which was the leading term of two groups, group 17 of which 36 GO terms belong and group 18, of which 55 GO terms belong, followed by “myeloid cell activation involved in immune response” (GO:0002275; p-value 2.02e-09) of which 13 GO terms belong, followed by “extracellular exosome (GO:0070062; p-value 2.00e-08) of which 8 GO terms belong. The leading term of all 19 groups can be found in **Figure 2** and full details of the GO term analysis can be found in **Supplementary Table 5**. The results of the network analysis showed that the significant GO terms form a dense integrated network of functional groups (**Figure 3**). Notable transcripts related to macrophage differentiation and/or function, that were DE in Day 1 and Day 5 HKLs and appeared in multiple GO terms, include *irf7* and *irf8* (both upregulated in Day 5 HKLs compared to Day 1 HKLs), *klf2* (downregulated in Day 5 HKLs compared to Day 1 HKLs), *csf1r* (upregulated in Day 5 HKLs compared to Day 1 HKLs), *arg1* (downregulated in Day 5 HKLs compared to Day 1 HKLs) and *fasn* (upregulated in Day 5 HKLs compared to Day 1 HKLs). The appearance of these DE transcripts in multiple GO terms that are associated with leukocyte differentiation and function (e.g. “innate immune response”, “leukocyte activation”, “hemopoiesis”, to name a few) provides evidence that these transcripts are important for these processes in Atlantic salmon adherent HKLs.

**Figure 4:**
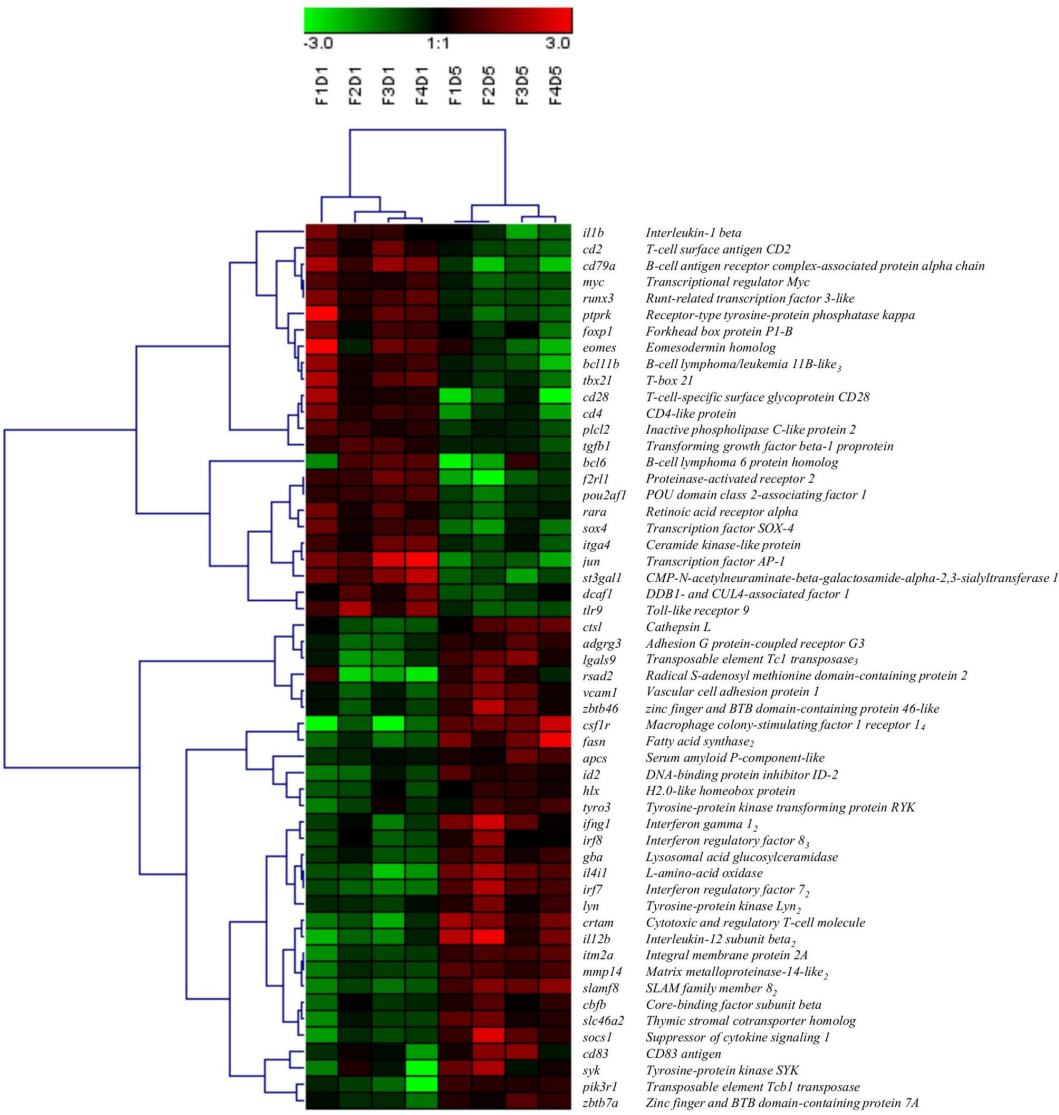
Hierarchical clustering analysis of DEGs associated with “mononuclear cell differentiation” (GO:1903131), shown as a heatmap. DEGs were median-centred and clustered using Pearson correlation and complete linkage hierarchical clustering. An average expression is shown when multiple probes were identified for one gene, and the subscript after the gene description indicates the number of probes. F indicates fish number; D indicates Day 1 or Day 5 (i.e. F1D1 is Fish 1 Day 1).

A total of 54 DEGs contributing to the GO term “mononuclear cell differentiation” (GO:1903131) were used for hierarchical clustering and displayed using a heat map (**Figure 4**). Similar to the clustering of all DEPs (**Figure 1B**), within the transcripts associated with the GO term “mononuclear cell differentiation”, all Day 1 samples clustered together, and Day 5 samples clustered together, indicating Day 1 and Day 5 samples consist of two groups of cells with distinct molecular phenotypes. Of the DE transcripts annotated with the GO term “mononuclear cell differentiation”, 44% were downregulated (e.g. *il1b, jun, cd28, cd4*) and 56% were upregulated (e.g. *csf1r, irf7, ifng1, fasn*) in Day 5 HKLs compared to Day 1 HKLs, suggesting that these transcripts are likely important in mononuclear cell differentiation in Atlantic salmon HKLs.

### RT-qPCR of DE Transcripts Validated Microarray Results

**Figure 5:**
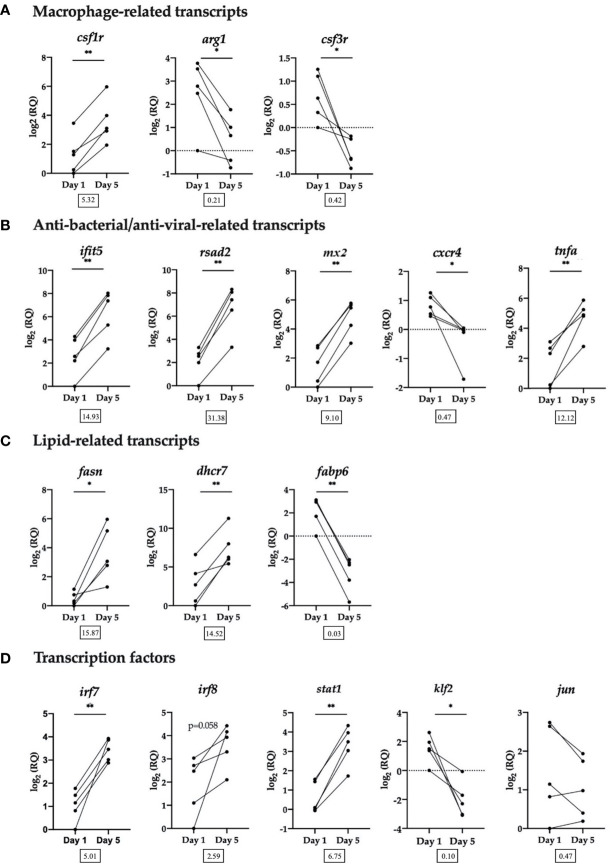
RT-qPCR validation of selected transcripts. **(A)** Macrophage-related transcripts. Colony-stimulating factor 1 receptor (*csf1r*), arginase-1 (*arg1*), granulocyte colony-stimulating factor receptor (alias colony stimulating factor 3 receptor (*csf3r))*. **(B)** Anti-bacterial/anti-viral-related transcripts. Interferon-induced protein with tetratricopeptide repeats 5 (*ifit5)*, radical SAM domain-containing 2 (*rsad2*, alias viperin), interferon-induced GTP-binding protein Mx (*mx2)*, C-X-C chemokine receptor type 4 (*cxcr4*), tumor necrosis factor alpha (*tnfa*). **(C)** Lipid-related transcripts. Fatty acid synthase (*fasn*), 7-dehydrocholesterol reductase (*dhcr7*), gastrotropin (alias fatty acid binding protein 6 (*fabp6)*). **(D)** Transcription factors. Interferon regulatory factor 7 (*irf7*), interferon regulatory factor 8 (*irf8*), signal transducer and activator of transcription 1 (*stat1*), krueppel-like factor 2 (*klf2*), transcription factor AP-1 (alias jun proto-oncogene (*jun)*). Data from each individual fish shown as log_2_(RQ), n = 5, *p < 0.05; **p < 0.01. The number under each figure represents the average fold-change in Day 5 HKLs compared to Day 1 HKLs.

Sixteen DE transcripts identified by the microarray were chosen for RT-qPCR validation. Transcripts were selected for RT-qPCR based on functional categories: macrophage-related transcripts, anti-bacterial/anti-viral-related transcripts, lipid-related transcripts and transcription factors (**Figure 5**).

The RT-qPCR results for all transcripts examined validated the microarray results, with the exception of *irf8*, which followed the same upregulated trend, but was not significant (p=0.058) and *jun* which followed the same downregulated trend but was not significant (p=0.164). In addition, using the same group of Atlantic salmon used in this RT-qPCR experiment, we previously confirmed a significant upregulation in Day 5 cells compared with Day 1 cells of two macrophage-related transcripts that were not identified as DE by the microarray but are known macrophage markers in the literature (*marco* and *MHC II*) (32). Of the transcripts examined by RT-qPCR, *rsad2* had the largest significant upregulated fold-change (FC) (FC = 31.38) in Day 5 HKLs, while *irf7* had the smallest significant upregulated FC (FC = 5.01). *Fabp6* had the largest significant downregulated FC (FC = 0.03) in Day 5 HKLs and *cxcr4* had the smallest significant downregulated FC (FC = 0.47).

### *In Silico* miRNA Target Gene Predictions and Target Gene Pathway Enrichment Analysis

Out of the 1477 DEGs identified in this current study, 1234 (84%) had 3’UTR information and could be included in the target prediction analysis. The analysis identified 680 of them to be potential targets of one, or more, of the 36 DE miRNAs selected from our previous comparison of miRNA expression in Day 1 monocyte-like cells and Day 5 macrophage-like cells in (32) (**Supplementary Table 6**). The gene pathway enrichment analysis shown in **Supplementary Table 7** identified gene pathways that were more likely to be regulated by miRNAs including interleukin-3, interleukin-5, and GM-CSF signaling; Fc gamma receptor-mediated phagocytosis; hematopoietic cell lineage; and lipid and lipoprotein metabolism. The complete overview of all pathways, p-values, and target genes participating in each pathway is given in **Supplementary Table 7**.

## Discussion

The aquaculture sector in Canada generates $5.4 billion CAD in economic activity annually (53). The Atlantic salmon is Canada’s top aquaculture product (by volume) and is therefore of high economic importance. Identifying how their immune cells develop and function is necessary to fully understand the fish immune system. HKLs have been used in many *in vitro* immunology studies involving several fish species [(27–31), among many others], but remain to be fully characterized. Our previous work observed a change in morphology, phagocytic ability, and miRNA profile of HKLs cultured for 5 days, from predominantly monocyte-like at Day 1 of culture to predominantly macrophage-like at Day 5 of culture (32). Several mammalian studies have observed large numbers of differentially expressed transcripts during monocyte-to-macrophage differentiation and/or macrophage polarization using high-throughput profiling methods, such as microarrays, many of which were identified in this current study and are discussed below (54–58). Therefore, we used a 44K microarray to examine changes in transcript expression profiles between Day 1 monocyte-like HKLs and Day 5 macrophage-like HKLs. Changes in the transcript expression of immune-related genes, lipid-related genes, and genes encoding transcription factors that are involved with macrophage differentiation, polarization, and function in other vertebrates were identified. In addition, GO term analyses identified biological processes including leukocyte differentiation, hematopoiesis, innate immune response and lipid metabolic process.

### Transcriptional Changes Associated With Macrophage Differentiation, Polarization, and Immune Response in Atlantic Salmon HKLs

The results of this study identified several macrophage and immune-related transcripts in both Day 1 and Day 5 HKLs. As the sample materials used in this study were immune cells, some of the identified transcripts were not unexpected. The paired SAM analysis identified differentially expressed transcripts between Day 1 and Day 5 HKLs that are involved in macrophage differentiation (including *csf1r* and *csf3r*), polarization of M1/M2 macrophages (including *arg1* and *ifng1*), and macrophage function (including *mx1, mx2* and *tlr3*).

The differentiation, proliferation, and survival of myeloid cells depends on signals derived from CSF1 upon binding with its receptor CSF1R (59–61). In humans and mice, CSF1R increases during macrophage differentiation, with CMPs expressing the lowest levels of CSF1R, monocytes expressing significantly more CSF1R and macrophages expressing the highest levels of CSF1R [reviewed in 52]. On the other hand, signaling through the granulocyte colony-stimulating factor 3 receptor (CSF3R, also known as GCSFR) is important for the proliferation, differentiation, and activation of neutrophils (62–64). Both *csf1r* and *csf3r* sequences have been identified in multiple fish species, and studies have indicated a conserved function for both receptors (4). As in mammals, *csf1r* has been identified as a marker of monocytes and macrophages in fish, and its expression is increased with macrophage differentiation (62, 65, 66). Similarly, *csf3r*, has been demonstrated to be necessary for neutrophil development in several fish species (67–69). In the current study, *csf1r* was significantly increased in Day 5 HKLs compared to Day 1 HKLs, while *csf3r* was significantly decreased in Day 5 HKLs compared to Day 1 HKLs, suggesting that, without the addition of exogenous factors, such as M1 (i.e. IFN-γ) and M2 (i.e. IL-4) activation stimuli, HKLs differentiate along the monocyte/macrophage lineage and not toward the granulocyte lineage during *in vitro* culturing. However, the downregulation of *csf3r* may also indicate that neutrophils were present at Day 1 of culture but had died off by Day 5. Several other transcripts related to different immune cells, including B cells (*cd79a, ighm, igha2, cxcr3*) and T cells (*cd2, cd4, cd8b, cd28, cd96*), were also downregulated in Day 5 cells compared to Day 1 cells (70–72). These results suggest that the Day 1 culture contained a heterogeneous mixture of several cell types but by Day 5 most of these cells were no longer present, leaving the Day 5 culture with a more homogenous population of cells (i.e. macrophages).

M1 “pro-inflammatory” macrophages and M2 “anti-inflammatory” macrophages can be defined based on their gene and protein expression profiles. Arginase enzyme activity and mRNA expression are hallmarks of M2 macrophages in both mammals and fish [reviewed in (3, 73)]. Like mammals, fish possess two arginase genes, *arginase-1* (*arg1)* and *arginase-2* (*arg2*) (3). While *arg1* is a marker of M2 macrophages in mammals, results have shown that *arg2* expression is a marker for the M2 phenotype in fish (19, 21, 74). Similarly, the chemokine receptors *cxcr1* and *cxcr4* are upregulated following M2 stimulation and are potential markers of M2 macrophages in mammals (*cxcr1* and *cxcr4)* and fish (*cxcr1)* (21, 54). This current study revealed a decrease in *arg1*, *cxcr1*, and *cxcr4* expression in Day 5 HKLs compared to Day 1 HKLs. Interestingly, we found a decrease in *arg1* expression (similar to mammals) and not *arg2* expression (similar to fish) in Day 5 HKLs, suggesting that the role of the arginase genes in macrophage differentiation and function may be species-specific. However, an examination of both *arg1* and *arg2* expression in Atlantic salmon, along with arginase enzyme activity in response to M2 stimulation, will be required to determine this.

Several markers of M1 macrophages, such as *tnfa, il12b*, and *ifng1* were upregulated in unstimulated Day 5 HKLs. These genes have been identified in different fish species and their role in the fish macrophage immune response and M1 polarization are conserved with other vertebrates (1, 3, 9, 75–77). On the other hand, markers of M2 macrophages in mammals, including *mrc1, socs1*, and *tgm*, were also upregulated in Day 5 HKLs. While these genes are present in fish, they have yet to be characterized as teleost M2 markers, unlike the M1 markers identified here (1, 3). It is interesting to find an upregulation of both M1 and M2 markers in non-stimulated cells. These results may indicate that during the culture, adherent HKLs become primed to develop into M1 or M2 macrophages upon stimulation. Future research, using functional studies with M1 and M2 activating stimuli, as well as protein expression data, would help to determine if the transcripts identified here are in fact M2 markers in teleost fish, as they are in mammals, and if the HKLs cells become primed to develop into the M1 or M2 phenotype during culture time.

In addition to the classic markers of macrophages, this study showed the differential expression of several virus-responsive, bacteria-responsive and inflammation-related genes in the two cell populations including Toll-like receptor 3 *(tlr3)*, interferon-induced GTP-binding proteins *mx1* and *mx2*, radical SAM domain-containing 2 *(rsad2*), interferon-induced protein with tetratricopeptide repeats 5 *(ifit5b)*, DExD/H-box helicase 58 (*ddx58*; also known as RIG-I), granulin (*grn*), hepcidin *(hamp)*, and legumain (*lgmn*). These genes have been described in many fish species and have similar immune-related functions as their mammalian counterparts (24, 27, 78–83). In mammals, *Tlr3* levels are highest in macrophages, compared to other mononuclear cells, and is not detected in neutrophils (84–86). While *tlr3* has been described in several fish species, it is unknown if *tlr3* is involved in HKL differentiation in fish. However, our results showed an upregulation of *tlr3* in Day 5 HKLs, suggesting that *tlr3* could be a novel marker of macrophages in fish. Legumain (*LGMN*) is associated with M2 macrophages (87–89) and its expression and activity is increased during monocyte-to-macrophage differentiation in both human THP-2 cells and murine RAW264.7 cells (88, 89). In goldfish, *lgmn* expression is highest in macrophages, compared to monocytes and progenitor cells, suggesting that *lgmn* may be a marker for macrophages in fish (24). Similarly, granulin may play a role in fish myeloid cell differentiation; in mutant zebrafish that do not express granulin, decreased differentiation of myeloid precursors into neutrophils and macrophages was observed, while adult mutants developed a head kidney with increased progenitors and decreased mature myeloid cells (90, 91). The upregulation of *lgmn* and *grn* in Day 5 HKLs, like the transcripts discussed thus far, point to the differentiation of HKLs into macrophages. In addition, the upregulation of virus-related and bacteria-related transcripts may indicate that Day 5 HKLs are more prepared to combat pathogen infection than Day 1 HKLs.

While we observed upregulation of several immune-relevant transcripts in Day 5 HKLs, there were also some immune-related genes (e.g. virus-responsive and bacteria-responsive) that were downregulated in Day 5 compared with Day 1 HKLs. For example, *il1b, tnfrsf6b, tnfrsf11b* and *tlr9* were downregulated in Day 5 HKLs compared to Day 1 HKLs. These genes have been demonstrated to be responsive to bacterial and viral challenges in various fish species (92–94). The upregulation of some pathogen-responsive genes, and the downregulation of others, in Day 5 HKLs compared with Day 1 HKLs, suggests that these cells are likely changing in their responsiveness to pathogens over time in culture. Future research should use live pathogen challenges at different time points during differentiation to test this hypothesis.

### Transcriptional Changes Associated With Lipid Metabolism Observed in Atlantic Salmon HKLs

Lipids play a major role in regulating many biological processes including cell growth, proliferation, and function. Lipids and fatty acids are required for a cell to grow and proliferate and, therefore, the enzymes involved in the formation of fatty acids are necessary for the development and differentiation of macrophages (95). Significant changes in the lipid-related transcriptome occur during mammalian monocyte-to-macrophage differentiation and M1/M2 polarization (57, 58, 96). Transcripts involved with fatty acid synthesis, elongation and desaturation, and cholesterol production, utilization and export are differentially expressed between mammalian monocytes and macrophages, as well as between M1 and M2 macrophages (57, 58, 96). In this study, transcripts related to the synthesis of fatty acids [e.g. fatty acid synthase (*fasn*) and long-chain fatty acid elongase 6 (*elovl6*)], transcripts involved in hydrolyzing triglycerides into free fatty acids [e.g. lipoprotein lipase (*lpl*)], and transcripts involved in fatty acid desaturation [e.g. fatty acid desaturase 2, *fads2*, alias delta-6 fatty acyl desaturase, *fadsd6;* and *fads1*, alias delta-5 fatty acyl desaturase*, fadsd5*)] were upregulated in Day 5 HKLs compared to Day 1 HKLs. In addition, GO term analysis identified lipid-related GO terms including lipid biosynthetic process (GO:0008610), neutral lipid catabolic process (GO:0046461) and cholesterol metabolic process (GO:0008203).

*FASN* is necessary for macrophage function in humans and the expression of both *FASN* and *Elovl6* is upregulated in human and mouse macrophages, respectively, upon differentiation from monocytes (57, 58, 96). While both *fasn* and *elovl6* have been described in numerous fish species, their role in macrophage differentiation in fish is unknown. In several fish studies, liver *fasn* and *elovl6* expression were found to be responsive to diet (97–100). In white Pacific shrimp (*Litopenaeus vannamei*), *fasn* expression was increased in the gills and hemocytes (immune cells of shrimp) following *V*. *parahaemolyticus* infection and knockdown of *fasn* increased morbidity, suggesting that *fasn* may have a role in immune cell response in some aquatic species; however, this requires further investigation (101).

Lipoprotein lipase is an enzyme that hydrolyzes triglycerides in lipoproteins found in chylomicrons and very low-density lipoproteins (VLDLs) into free fatty acids. A dramatic upregulation of *LPL* was observed in human macrophages differentiated with M-CSF, as well as without exogenous factors (58, 96). Furthermore, differentiation of bone marrow cells from LPL-deficient mice had 40% less differentiated macrophages than control mice, suggesting that LPL is necessary for macrophage differentiation (102). Like *fasn* and *elovl6*, *lpl* expression in fish has been reported to be modified by diet (103–105), however, its role in HKLs differentiation and/or function is unknown. The increased expression of *fasn*, *elovl6*, and *lpl* suggests the need for macrophages to access fatty acids for inflammatory functions and this need is conserved in fish and mammals. Additionally, the high expression level of these transcripts may serve as novel markers of macrophages in fish.

Fatty acid desaturases are enzymes required for the synthesis of omega-3 and omega-6 polyunsaturated fatty acids (PUFAs) through the formation of double bonds between fatty acyl chain carbons. Fatty acid desaturase 2 (*fads2*, alias *fadsd6*) and fatty acid desaturase 1 (*fads1*, alias *fadsd5*) were upregulated in Day 5 HKLs compared to Day 1 HKLs. While most studies to date have examined *fads2* and *fads1* expression in organs with high fatty acid turnover, such as the liver, there are data suggesting that they play a role in myeloid cells (106, 107). In human macrophages, *FADS2* expression increased during monocyte-to-macrophage differentiation and inhibition of FADS2 in human peripheral blood mononuclear cells (PBMCs) decreased the number of proliferating cells. Similar to *fasn, elovl6*, and *lpl, fads2* and *fads1* have been described in fish species (108), and their expression level in HKLs is regulated by nutrition and diet (109–113). The role of *fads2* and *fads1* in HKL differentiation and function has not been investigated, however, the results of this study suggest that, along with *fasn and lpl, fads2* and *fads1* may be conserved markers of macrophages and macrophage function.

7-dehydrocholesterol reductase (DHCR7) is an enzyme that catalyzes the production of cholesterol in the final step of cholesterol biogenesis (114). A significant increase in *dhcr7* was observed in Day 5 HKLs compared to Day 1 HKLs, suggesting an increase in cholesterol biosynthesis in Day 5 HKLs. Ecker et al. (57) observed an increase in *DHCR7* expression in primary human monocytes undergoing macrophage differentiation for 4 days. Interestingly, the increase in *DHCR7* expression at 4 days, decreased to below baseline (day 1) values following 6 days of macrophage differentiation. Similar to the lipid-related transcripts discussed here, liver, muscle, and gut *dhcr7* is responsive to diet in several fish species, but the role of *dhcr7* in macrophage differentiation and/or function in fish has yet to be investigated (115–117).

### Transcription Factors Involved in Mammalian Macrophage Differentiation Were DE in Atlantic Salmon HKLs

Macrophage differentiation and polarization are tightly regulated by transcription factors (TFs) and are associated with large changes in transcriptional programming. The TFs that regulate myeloid cell differentiation and macrophage polarization have been extensively studied and characterized in mammals, while this area of research is expanding in teleost fish (4, 23, 118). Transcripts encoding several TFs involved in mammalian macrophage biology were differentially expressed in Day 1 and Day 5 HKLs in the current study, suggesting possible conserved roles for these TFs. In the present study, members of the Krueppel-like factors (KLF) family (i.e. *klf2, klf9*) were downregulated in Day 5 HKLs compared to Day 1 HKLs, while members of the interferon regulatory factor (IRF) family (i.e. *irf3*, *irf7*, *irf8*), as well as signal transducer and activator of transcription 1 (*stat1*), were upregulated in Day 5 HKLs compared to Day 1 HKLs.

KLFs are members of the zinc-finger family of TFs which play roles in many biological processes including cell proliferation, differentiation, growth, apoptosis, and inflammation (119, 120). In primary human monocytes, *KLF2* expression is reduced upon differentiation into macrophages and its overexpression in the THP-1 human cell line inhibited LPS-induced cytokine secretion and decreased phagocytic ability, indicating that the suppression of KLF2 is necessary for macrophage differentiation and function (121). Similarly, KLF9 overexpression in RAW264.7 murine cell line reduced LPS-induced inflammatory cytokine release (122). While KLF9 is mostly known for its involvement in B-cell differentiation (123), these studies suggest that KLF2 and KLF9 have a role in monocyte maintenance and their downregulation is necessary for macrophage differentiation. This current study found a decrease of both *klf2* and *klf9* in Day 5 HKLs compared to Day 1 HKLs. There is very little information on fish KLFs, however, there are recent studies that provide evidence for a role of KLF2 and KLF9 in the immune response (124, 125). For example, KLF2 expression was found to be highest in PBMCs of ayu (*Plecoglossus altivelis*) compared to other tissues (liver, spleen, brain, gill, head kidney) and its expression increased with *L. anguillarum* infection. Furthermore, siRNA knockdown of KLF2 increased *il1b* and *tnfa* expression in both resting and *L. anguillarum* infected head kidney monocytes/macrophages, suggesting that, similar to mammalian cells, KLF2 suppresses ayu monocyte/macrophage activation (124). While the role of KLF2 and KLF9 in macrophage differentiation and polarization is unknown in fish, the results of this study suggest that, as in mammals, these TFs are involved in regulating myeloid cell differentiation in fish. It is possible that KLF2 and/or KLF9 play a role in maintaining the monocyte or precursor population and their decrease in expression is necessary for macrophage differentiation and function.

Members of both the IRF and STAT TF families have been implicated in a wide range of cellular events, including cell growth, proliferation, survival, and immune responses and each has members that are important mediators of macrophage polarization and/or differentiation (126). IRF3, IRF7 and IRF8 are involved in mammalian macrophage differentiation, polarization and/or function (127). The expression of both IRF8 and IRF7 increases during macrophage differentiation, while the expression of IRF8 declines upon granulocytic differentiation (128–130). Furthermore, IRF8 is necessary for the formation of mature, functional macrophages while the expression of IRF7 is both necessary and sufficient to induce monocyte-to-macrophage differentiation in U937 monocytic cell line (128–130). In mammals, IRF3 is associated with M1 polarization (131, 132). In fish, *irf8* is specifically associated with primary macrophages during zebrafish embryogenesis (90). While *irf8* null mutants have decreased macrophage development and enhanced neutrophil production, overexpression of *irf8* in the mutants could partially recover this effect (90). Similar to mammals, both *irf3* and *irf7* are responsive to viral infection in a fish monocyte/macrophage cell line (RTS11), as well as primary fish macrophages, suggesting that *irf3* and *irf7* have a role in the immune response of fish macrophages (133, 134). The increase in *irf3, irf7* and *irf8* expression in Day 5 HKLs compared to Day 1 HKLs observed in the current study may indicate that, if the functions of these genes are the same in fish as they are in mammals, then the Day 5 culture is composed more of macrophages compared to the Day 1 culture.

In primary human monocytes, STAT1 activity increased as monocytes differentiated into macrophages (135). Moreover, STAT1 binding was detected in the promoters of genes important for macrophage differentiation and function, such as *FcyRI*, *ICAM-1* and *IRF1* (135). In several fish species, *stat1* expression and/or signaling, as well as M1 markers, are increased in head kidney leukocytes following IRF-γ stimulation (115, 116, 136). Here we found an upregulation of *stat1* in Day 5 HKLs compared to Day 1 HKLs, suggesting an increase in *stat1* is indicative of macrophage differentiation in the Day 5 culture.

### DE miRNAs Are Predicted to Target DE Transcripts and Are Associated With Macrophage Immune Function Gene Pathways

miRNAs are short, non-coding RNAs that play a role in regulating gene expression by binding to a partially complementary sequence in the (usually) 3’ UTR of their target mRNA, leading to mRNA degradation or the prevention of translation (137). miRNAs regulate several biological processes including cell differentiation and immune response, among many others [reviewed in (138, 139)]. Work in mammals has demonstrated that miRNAs can mediate the differentiation and activation of macrophages (140, 141). Our previous work identified 66 DE miRNAs when comparing Day 1 and Day 5 HKLs (22 miRNAs downregulated and 44 miRNAs upregulated in Day 5 HKLs, compared to Day 1 HKLs) (32), including many that are involved in mammalian macrophage function (e.g. miR-146a, miR-155 and miR-21) (142–144), as well as teleost fish immune response (e.g. miR-146a, miR-462, miR-2188 and miR-731) (145, 146). The 36 major expressed DE miRNAs (32), likely to be the biologically relevant guide-miRNAs, were used as input against the 3’UTRs from the DEGs identified in the current study. This targeted approach could identify whether any of the DEGs are potential targets of the DE miRNAs in (32). This is a first step to determine which miRNAs may be involved in monocyte-to-macrophage differentiation by targeting DEGs for post-transcriptional regulation by the RISC-complex.

The results from the *in silico* target prediction applying the selected DE miRNAs from (32) and all DEGs with 3’UTR information revealed that 660 of the DE transcripts identified in the current study were potential targets. It is unlikely that more than half of the DEGs are true targets as there are usually a large percentage of false positives for several reasons in these predictions (147). However, such *in silico* predictions are still used as a first means to single out which DEGs may be true miRNA targets. Among the interesting putative targets with known roles in macrophage differentiation and/or function with predicted miRNA response elements for particular DE miRNAs were *tnfa* (ssa-miR-214-1-3p and ssa-miR-139-5p), *fadsd5* (ssa-miR-21a-5p), and *ifit5* (ssa-miR210-1-5p and ssa-miR-22a-3p), all of which showed increased expression in Day 5 cells. Other interesting predicted targets like *arg1* (ssa-miR-214-3-3p and ssa-miR-2188-3p)*, cxcr4* (ssa-miR-214-3-3p)*, klf2* (miR-181a-5p, ssa-miR-29b-3p and ssa-miR-novel-16-5p)*, klf9* (ssa-miR-155-5p, ssa-miR-214-3-3p and ssa-miR-210-1-5p), and *il1b* (ssa-miR-139-5p, ssa-miR-24ac-3p and ssa-miR-725-3p) all showed decreased expression in Day 5 cells. The traditionally acknowledged function of miRNAs is to downregulate gene expression which would lead to a decrease of target transcripts if the miRNA expression increases (137). Such inverse relationships were not always the case between a miRNA and its predicted target from our *in silico* analysis. However, the function of most cellular miRNAs is to maintain equilibrium of the target transcripts, which is regulated positively by the rate of transcription and negatively by miRNAs. Differentiation of a cell type that is dependent on an increased level of a given transcript can be triggered by transcriptional activation. However, the miRNAs that contribute to maintaining this transcript in equilibrium would also increase in order to maintain the higher expression level of this transcript in balance. Such relationships between a miRNA and its target, also referred to as feed forward loops (148), lead to increases of both the targets and their miRNAs as they are (often) activated by the same transcription factors. Similar dynamics have been proposed for miRNAs associated with immune responses and their targets (147), and many of the DE miRNA genes changing expression in Day 5 HKLs have upstream transcription binding motifs of *irf8*, *irf1*, and *irf3* (146) that are increased in Day 5 HKLs in this study. Future functional studies, using knock-out or overexpression models, are required to fully determine if a DE gene identified in this study is the target of a certain DE miRNA identified in our previous work (32).

The DEGs found in this current study that were identified as potential targets (see **Supplementary Table 6**) of the DE miRNAs (32) were used for pathway enrichment analysis. The results showed that the putative target genes were significantly enriched in pathways associated with macrophage immune function, such as interleukin-3, interleukin-5, and Fc gamma receptor-mediated phagocytosis, pathways associated with macrophage differentiation, such as GM-CSF signaling and hematopoietic cell lineage, and lipid-related pathways such as lipid and lipoprotein metabolism. Although not proving certain miRNA-target interactions, the enrichment of these gene pathways further suggests that the miRNAs are involved in macrophage maturation.

## Conclusion

The aim of the current study was to build on our previous work (32) and examine changes in gene expression of Atlantic salmon HKLs *in vitro*. We identified immune-related transcripts, lipid-related transcripts, and transcripts encoding TFs that were differentially expressed between Day 5 and Day 1 HKL populations. Many of the identified transcripts are markers of macrophages, involved in M1/M2 polarization and/or involved in macrophage function in other species, suggesting a conserved function for some of the transcripts, as well as the possibility of using these transcripts as macrophage markers, although future functional studies are required to confirm this. Overall, the results indicate that, without the addition of exogenous factors, the HKL cell population differentiates *in vitro* to become macrophage-like, and this dynamic change in cell population is an important consideration when working with Atlantic salmon HKLs *in vitro*.

## Data Availability Statement

The datasets presented in this study can be found in online repositories. The names of the repository/repositories and accession number(s) can be found in the article/**Supplementary Material**.

## Ethics Statement

The animal study was reviewed and approved by Memorial University of Newfoundland’s Institutional Animal Care Committee.

## Author Contributions

Conceptualization: NS, SC, and MR. Methodology: NS, SC, and MR. Software: NS, NU, SK, NW, and RA. Validation: NS. Formal analysis: NU, SK, NW, and RA. Investigation: NS. Resources: SC and MR. Data curation: NS, NU, SK, NW, and RA. Writing—original draft preparation: NS. Writing—review and editing: NS, NU, SK, NW, RA, SC, MR. Visualization: NS. Supervision: SC and MR. Project administration: NCS, SC, and MR. Funding acquisition, SC and MR. All authors contributed to the article and approved the submitted version.

## Funding

This study was funded by Natural Sciences and Engineering Research Council of Canada (NSERC) Discovery Grants to MR (341304-2012 and 2020-04519), a Memorial University of Newfoundland Seed grant to SC (212779), a NSERC Discovery Grant to SC (2017-04630), a Norwegian Research Council grant to RA (280839/E40), and Genomic Applications Partnership Program projects [GAPP # 6604: Biomarker Platform for Commercial Aquaculture Feed Development project; and GAPP #6607: Integrated Pathogen Management of Co-infection in Atlantic salmon (IPMC) project] funded by the Government of Canada through Genome Canada and Genome Atlantic, and Cargill Innovation (formerly EWOS Innovation) to MR. The IPMC project was also funded by the Government of Newfoundland and Labrador through the Department of Tourism, Culture, Industry and Innovation (Leverage R&D award #5401-1019-108. NS was supported by a NSERC PGS D fellowship.

## Conflict of Interest

The authors declare that the research was conducted in the absence of any commercial or financial relationships that could be construed as a potential conflict of interest.

## Publisher’s Note

All claims expressed in this article are solely those of the authors and do not necessarily represent those of their affiliated organizations, or those of the publisher, the editors and the reviewers. Any product that may be evaluated in this article, or claim that may be made by its manufacturer, is not guaranteed or endorsed by the publisher.

## Supplementary Material

The Supplementary Material for this article, including details on qPCR assays used [27, 32, 149–151] can be found online at: https://www.frontiersin.org/articles/10.3389/fimmu.2021.709910/full#supplementary-material
